# Spectrum of autoantibodies and other serological parameters in connective tissue disease–associated interstitial lung disease patients

**DOI:** 10.3389/fmed.2026.1812588

**Published:** 2026-06-03

**Authors:** Pooja Jaiswal, Tanya Athavale, Amita Athavale, Hemangini Thakkar, Ridi Khatri, Amrutha Jose, Trisha Samant, Tanaya Tipnis, Namrata Neman, Milind Nadkar, Anjali Rajadhyaksha, Manisha Madkaikar, Vandana Pradhan

**Affiliations:** 1 Department of Clinical & Experimental Immunology, Indian Council of Medical Research, National Institute of Immunohaematology, Mumbai, India; 2 Department of Pulmonary Medicine and Environmental Pollution Research Centre, Seth G. S. Medical College and King Edward Memorial Hospital, Mumbai, India; 3 Department of Radiology, Seth G. S. Medical College and King Edward Memorial Hospital, Mumbai, India; 4 ICMR-National Institute of Immunoheamatology, Mumbai, India; 5 Department of Medicine, Seth G. S. Medical College and King Edward Memorial Hospital, Mumbai, India; 6 Department of Paediatric Immunology and Leukocyte Biology, ICMR-National Institute of Immunohaematology, Mumbai, India

**Keywords:** ANA specificities, autoantibodies, connective tissue diseases (CTD), CTD-ILD, interstitial lung disease (ILD)

## Abstract

**Background:**

Connective tissue disease–associated interstitial lung disease (CTD-ILD) is a major cause of morbidity and mortality in autoimmune disorders, yet detailed serological characterization remains limited in Indian cohorts.

**Objective:**

This study aimed to evaluate and compare demographic, radiological, autoantibody, and serum profiles between CTD-ILD and non-CTD-ILD patients from Western India and to delineate hospitalization patterns and mortality outcomes among CTD-ILD patients.

**Methods:**

This single-centre, cross-sectional study enrolled 200 patients with ILD (*n* = 90 with CTD-ILD and *n* = 110 with non-CTD-ILD) from a tertiary care centre who were evaluated on a clinico-radiological basis. Antinuclear antibodies and ANA subspecificities were assessed using indirect immunofluorescence and a line blot assay. Serological parameters were quantified using ELISA and multiplex bead-based assays.

**Results:**

CTD-ILD patients were significantly younger (49 vs. 57 years; *p* < 0.001) and predominantly female (80.0% vs. 59.1%; *p* = 0.002). NSIP predominated in CTD-ILD (51.1%), whereas UIP predominated in non-CTD-ILD (46.4%; *p* < 0.001). On multivariable analysis, younger age (aOR = 0.954; *p* < 0.001), female sex (aOR = 2.709; *p* = 0.023), NSIP (aOR = 6.155; *p* = 0.001), and UIP (aOR = 8.877; *p* < 0.001) were independent predictors of CTD-ILD. ANA positivity was higher in CTD-ILD (70.0% vs. 54.5%; *p* = 0.025), with a predominance of high-titre ANA (81.0%, ≥1:160) and a speckled IFA pattern. The nucleolar pattern was significantly more prevalent in the CTD-ILD group (11.1%; *p* = 0.010). Disease-specific autoantibody enrichment was observed as follows: anti-SSA/Ro and AMA-M2 in RA-ILD, anti-RNP/Sm in MCTD-ILD, and anti-Scl-70 in SSc-ILD. Anti-MDA5 (*p* = 0.001), LDH (*p* = 0.019), and pro-inflammatory cytokines TNF-α, IFN-γ, IL-4, and IL-22 were significantly elevated in CTD-ILD, whereas MMP7 levels were lower (*p* = 0.025). TNF-α, IL-22, and IL-4 remained independent predictors in the multivariable analysis. Among CTD-ILD patients, 20.0% required hospitalization, with significantly higher in-hospital mortality in admitted patients (27.8% vs. 4.2%; *p* = 0.007) than in non-admitted CTD-ILD patients.

**Conclusion:**

CTD-ILD patients demonstrated distinct demographic, radiological, autoantibody, and serological profiles compared to non-CTD-ILD patients, characterized by elevated anti-MDA5, LDH, and pro-inflammatory cytokines alongside lower MMP7 levels, reflecting an immune-driven rather than fibrosis-dominant pathobiology. These findings contribute to bridging the knowledge gap in the serological characterization of CTD-ILD in Indian cohorts.

## Introduction

Interstitial Lung Disease (ILD), now referred to as Interstitial Pneumonias (IP), is a group of diverse lung disorders that primarily affect the lung interstitium and involve both inflammation and fibrosis (1). While many ILD cases are idiopathic, others arise from environmental or occupational exposures or from underlying conditions such as connective tissue disorders (CTD). The global prevalence of CTD-ILD ranges from 0.47 to 12.1 cases per 100,000 people (2). The occurrence of ILD varies across CTD subtypes, with systemic sclerosis (SSc) being the most frequent, followed by mixed connective tissue disease (MCTD) and idiopathic inflammatory myopathy (IIM), whereas rheumatoid arthritis (RA), Sjögren’s syndrome (SjS), and systemic lupus erythematosus (SLE) are less common (3). ILD is a leading cause of morbidity and mortality in CTD patients, particularly in fibrosing phenotypes. Five-year mortality rates of up to 39% in RA-ILD cohorts and 35% in myositis-ILD cohorts underscore the prognostic impact of progressive fibrosis (4, 5).

The diagnosis of CTD-ILD is complex, requiring a multidisciplinary framework that integrates clinical, radiological, and serological features. Autoantibodies are central to the diagnosis and prognosis of CTD-ILD, where antinuclear antibody (ANA) positivity, extractable nuclear antigen (ENA) specificities, and myositis-specific autoantibodies have been linked to disease subsets and outcomes (6). However, most international studies have reported only the presence of autoantibodies, with limited stratification of ANA titres and/or indirect immunofluorescence (IFA) patterns in CTD-ILD patients. This represents a diagnostic gap, particularly in resource-limited settings where multiplex assays are not routinely available.

Dysregulated extracellular matrix deposition is central to ILD pathogenesis and is driven by activated myofibroblasts. Matrix metalloproteinases (MMPs), a family of zinc-dependent endopeptidases, are central regulators of extracellular matrix (ECM) remodelling in ILD, mediating the balance between matrix degradation and aberrant fibrotic deposition (7). Among these, MMP1 initiates collagen degradation and promotes myofibroblast differentiation; MMP9 disrupts the alveolar basement membrane, facilitating inflammatory cell infiltration; and MMP7, produced by alveolar epithelial cells and macrophages, has been validated as a differential fibrosis marker, with significantly lower serum levels in CTD-ILD than in idiopathic pulmonary fibrosis (IPF). Tissue inhibitor of metalloproteinase-1 (TIMP-1), the principal endogenous MMP inhibitor, modulates the MMP/TIMP balance, with elevated levels associated with progressive fibrosis and adverse outcomes in ILD. Higher MMP7 and MMP9 levels have been reported in CTD-ILD patients, suggesting their utility in diagnosing ILD involvement in CTD patients (8). Krebs von den Lungen-6 (KL-6), a mucin-like glycoprotein overexpressed by damaged type II pneumocytes, directly reflects alveolar-capillary barrier disruption and correlates with HRCT fibrosis scores, pulmonary function impairment, and disease progression in CTD-ILD (9). Surfactant protein-D (SP-D), a collectin regulating innate pulmonary immunity, similarly reflects type II pneumocyte injury, and SP-D levels have been independently associated with SSc-ILD alongside KL-6, C-C motif chemokine ligand 18 (CCL18), and MMP7 (10). Their parallel evaluation provides complementary information on alveolar epithelial injury activity in CTD-ILD. Intercellular adhesion molecule-1 (ICAM-1), upregulated on the vascular endothelium by TNF-α and IFN-γ, mediates leukocyte transmigration into the pulmonary interstitium and has been incorporated into multivariate ILD progression models alongside MMP7 and osteopontin (11). Lactate dehydrogenase (LDH), released upon immune-mediated cellular necrosis, serves as an accessible marker of pulmonary cellular damage, with elevated levels associated with rapidly progressive disease and increased short-term mortality in anti-melanoma differentiation-associated gene 5 (anti-MDA5)-positive ILD (12). Serum ferritin, a surrogate marker of macrophage activation, is markedly elevated in IIM-ILD (13). Circulating immune complex-C1q (CIC-C1q) reflects complement-mediated immune complex deposition, a mechanism particularly relevant in SLE-ILD and vasculitis-associated ILD (14). Angiotensin-converting enzyme (ACE), produced by alveolar macrophages and endothelial cells, serves as an important differential diagnostic parameter for sarcoidosis-associated ILD (15). Anti-MDA5 antibodies, directed against a cytosolic innate immune RNA helicase, are strongly associated with amyopathic dermatomyositis-ILD, and anti-MDA5 titres alongside ferritin predict prognosis and treatment response in rapidly progressive ILD (16). The cytokine milieu in CTD-ILD reflects coordinated Th1/Th17/Th2 immune activation sustaining the immunity-to-fibrosis transition. Among Th1 cytokines, interleukin-1 beta (IL-1β) drives inflammasome-mediated macrophage activation; tumor necrosis factor-alpha (TNF-α) promotes epithelial-mesenchymal transition through NF-κB signalling, and interferon-gamma (IFN-γ) activates M1 macrophages, driving immune-mediated alveolar damage [cite]. Among Th17 cytokines, interleukin 6 (IL-6) bridges innate and adaptive immunity, driving Th17 differentiation and B-cell autoantibody production; interleukin-17A (IL-17A) recruits neutrophils and promotes fibroblast collagen synthesis; and interleukin-22 (IL-22) sustains aberrant alveolar epithelial remodelling (17). The Th2 cytokine interleukin-4 (IL-4) drives M2 macrophage polarization and fibroblast proliferation, with its downstream mediator CCL18 reaffirmed as a particularly robust predictor of progressive fibrotic disease in CTD-ILD. The simultaneous evaluation of cytokines across all three axes provides a comprehensive immunological fingerprint of CTD-ILD in the present study (10, 17).

Hospitalization and in-hospital mortality represent critical milestones in the clinical course of CTD-ILD. International cohorts have reported in-hospital mortality rates ranging from 10 to 30% in CTD-ILD patients, with the usual interstitial pneumonia (UIP) pattern on high-resolution computed tomography (HRCT), comorbidity burden, and impaired pulmonary function identified as key determinants of adverse outcomes (18). However, data on hospitalization patterns and mortality outcomes from Indian CTD-ILD cohorts remain scarce, limiting the understanding of disease burden in this population.

Regional Indian registries have reported prevalence data for CTD-ILD, but detailed serological characterization compared with non-CTD-ILD patients from this region remains limited (19, 20). Therefore, this study aims to evaluate and compare demographic, radiological, autoantibody, and serum parameter profiles between CTD-ILD and non-CTD-ILD patients from Western India and to delineate hospitalization patterns and mortality outcomes among CTD-ILD patients. By stratifying ANA IFA patterns by HRCT subtypes and integrating fibrosis markers, cytokines, and matrix remodelling parameters, this study further aimed to identify serological signatures specific to Indian CTD-ILD patients and to contribute to the global understanding of autoimmune-driven ILD.

## Materials and methods

### Study participants

This single-centre, cross-sectional study included 200 clinically diagnosed ILD patients from a tertiary care centre, Department of Pulmonary Medicine & Environmental Pollution Research, King Edward Memorial Hospital, Mumbai, India. These patients were enrolled between May 2022 and May 2025 at the ICMR-National Institute of Immunohaematology (ICMR-NIIH), Mumbai, India. The study was conducted after obtaining approval from the institutional ethics committees of both organizations, and patients were enrolled in both inpatient and outpatient departments after obtaining written informed consent. All CTD-ILD patients (*n* = 90) were evaluated by rheumatologists at the Department of Medicine, G. S. Medical College, and King Edward Memorial Hospital, Mumbai, India. These patients fulfilled the defined classification criteria proposed and updated by the American College of Rheumatology (ACR) and the European League against Rheumatism (EULAR) (21, 22).

The CTD-ILD group included rheumatoid arthritis-associated ILD (RA-ILD, *n* = 30), mixed connective tissue disease-associated ILD (MCTD-ILD, *n* = 8), idiopathic inflammatory myopathy-related ILD (IIM-ILD, *n* = 1), systemic sclerosis-associated ILD (SSc-ILD, *n* = 12), Sjögren’s syndrome-associated ILD (SjS-ILD, *n* = 3), systemic lupus erythematosus-associated ILD (SLE-ILD, *n* = 2), interstitial pneumonia with autoimmune features (IPAF, *n* = 5), unclassified CTD-ILD (*n* = 23), and ANCA-associated vasculitis [antineutrophil cytoplasmic antibody- associated vasculitis ILD (AAV-IL D), *n* = 6]. IPAF and unclassified CTD-ILD were distinguished based on the 2015 European research society/ American thoracic society (ERS/ATS) Task Force criteria (23), wherein IPAF was defined by the presence of at least one feature from at least two of three domains, *viz.,* clinical, morphological, and serological, in a patient with ILD who does not fulfil criteria for a defined CTD. ILD patients who demonstrated clinical and/or serological features suggestive of an underlying autoimmune process but did not fulfil the minimum two-domain requirement for IPAF, yet clearly could not be attributed to idiopathic or exposure-related causes, were classified as unclassified CTD-ILD following multidisciplinary rheumatological and pulmonological evaluation. The non-CTD-ILD group (*n* = 110) comprised ILD patients categorized as fibrotic hypersensitivity pneumonitis (fHP, *n* = 30), idiopathic NSIP (iNSIP, *n* = 22), idiopathic pulmonary fibrosis (IPF, *n* = 30), and other rare ILDs (*n* = 28). The ILD patients with clinical suspicion of any active infection, including COVID, tuberculosis, and neoplasm, were excluded.

### Baseline characteristics

Patients’ baseline demographic and clinical data, including age, sex, comorbidities, previous infection history (Koch’s disease), smoking history, and family history, were systematically recorded in case record forms (CRFs). Comorbidities assessed included diabetes mellitus (DM), hypertension (HTN), ischaemic heart disease (IHD), cerebrovascular accident (CVA), chronic kidney disease (CKD), and chronic liver disease (CLD). Treatment details of the use of non-steroidal anti-inflammatory drugs (NSAIDs), disease-modifying antirheumatic drugs (DMARDs) such as hydroxychloroquine (HCQ), mycophenolate mofetil (MMF), azathioprine (AZA), and methotrexate (MTX), and antifibrotics such as nintedanib and pirfenidone were also recorded. Hospital admission and in-hospital mortality were recorded for the enrolled patients during the study period.

### Radiological and pulmonary function test (PFT) parameters

High-resolution computed tomography (HRCT) and pulmonary function test (PFT) findings were documented in each patient, which were performed closest to the time of enrolment as part of routine clinical care. All HRCT scans were interpreted by radiologists and subsequently reviewed by the treating pulmonologists for clinical expertise. HRCT patterns were classified according to the ATS/ERS classification criteria, *viz.,* usual interstitial pneumonia (UIP), non-specific interstitial pneumonia (NSIP), and patterns other than UIP and NSIP were classified as “others.” PFT parameters recorded were calculated using the Indian standards (24) based on Global Lung Function Initiative (GLI) 2022 race-neutral reference equations, as recommended by the American Thoracic Society and European Respiratory Society. These equations were applied uniformly across all subjects, ensuring consistency and avoiding racial/ethnic bias in spirometry interpretation. These included forced vital capacity (FVC) as a percentage of actual to predicted values (FVC Act%), the ratio of actual to predicted FVC (FVC Act/Pred), forced expiratory volume in 1 s as a percentage of actual to predicted values (FEV₁ Act%), and the ratio of FEV₁ to FVC (FEV₁/FVC%).

### Autoimmune workup

Blood was collected in a plain vacutainer at a single time point at the time of enrolment. The serum was separated and stored at −80 °C until tested. The serum was used for testing various autoantibodies by indirect immunofluorescence assay (IFA) and the enzyme-linked immunosorbent assay (ELISA). The IFA technique for autoimmune workup was performed using HEp-2 cells, and Crithidia lucillae was used to screen for antinuclear antibodies (ANA) and anti-dsDNA autoantibodies, respectively. Antineutrophil cytoplasmic antibodies (ANCA) were tested by IFA (EUROIMMUN, Germany). ANA subspecificities for ANA-positive (>1:160) patients were tested using the LINE Blot assay (EUROIMMUN, Germany).

### Investigations of other serological parameters

The majority of immunological parameters, including MMP1, MMP7, MMP9, CCL18, KL-6/MUC1, ICAM, SP-D, anti-TPO, and TIMP1 levels, were quantified using commercially available ELISA kits (Elabscience Biotechnology Inc., Houston, Texas, USA). Inflammatory parameters such as ferritin, CIC-C1q, ACE, and hsCRP were tested using ELISA kits (Calbiotech lnc., California, USA; DRG lnc., USA; Bio-Techne R&D Systems lnc., Minnesota, USA; and Calbiotech lnc., California, USA, respectively). LDH, anti-MDA5 Ab, and VEGF-A levels were investigated using ELISA (SARD Biosciences LLP, Mumbai, Maharashtra, India). IL-1β, TNF-α, IFN-γ, IL-4, IL-6, IL-17A, and IL-22 levels were analysed using a multiplex bead-based immunoassay (AimPlex Biosciences Inc., California, USA), acquired using BD FACSDiva™ Software v8.0.1 (BD Biosciences, California, USA), and analysed using BD FCAP Array™ Software v3.0 (BD Biosciences, California, USA). All assays were performed in accordance with the manufacturer’s protocols.

### Statistical analysis

All statistical analyses were performed using IBM SPSS Statistics (version 20.0; IBM Corp., Armonk, NY, USA) and GraphPad Prism version 10.0.0 (GraphPad Software, San Diego, CA, USA). The normality of the data distribution was assessed using the Shapiro–Wilk test. Continuous variables with non-normal distribution were expressed as medians with interquartile range (IQR, 25th–75th percentiles) and were compared between groups using the Mann–Whitney *U* test. Categorical variables were expressed as frequency and percentage [*n* (%)] and were compared using Pearson’s chi-square test or Fisher’s exact test with continuity correction when the expected cell count was less than 5. Correlations between continuous variables were assessed using Spearman’s rank correlation coefficient. Multivariable binary logistic regression analysis was performed for serological parameters that demonstrated statistically significant differences between CTD-ILD and non-CTD-ILD patients on univariate analysis, adjusting for the following confounders: age, sex, radiological pattern (UIP, NSIP, and other patterns), and hypertension. Results were expressed as odds ratios (OR) with 95% confidence intervals (CI) and unadjusted and adjusted *p-*values. A two-tailed *p*-value of less than 0.05 was considered statistically significant for all analyses. Missing values in continuous variables were imputed using median imputation, applied independently within the CTD-ILD and non-CTD-ILD groups, for parameters with up to 20% missingness. Variables with greater than 20% missing data were not imputed and were analysed with available data only (25). The extent of missing data for each serological parameter is reported in Supplementary Table S1.

## Results

### Demographic, clinical, and radiological parameters

A total of 200 ILD patients were analysed, including 90 CTD-ILD and 110 non-CTD-ILD patients. Their demographic, clinical, and radiological details are presented in Table 1. CTD-ILD patients were predominantly female (80.0% vs. 59.1%, *p* = 0.002), whereas male patients were more frequent in the non-CTD-ILD group (40.9% vs. 20.0%, p = 0.002). CTD-ILD patients were significantly younger at the time of evaluation than non-CTD-ILD patients (49 years [IQR: 36–58 years] vs. 57 years [IQR: 47–68 years]; *p* < 0.001) and also demonstrated an earlier age of disease onset (45 years [IQR: 35–57 years] vs. 56 years [IQR: 47–68 years]; *p* < 0.001). The median disease duration was similar in both the CTD-ILD and non-CTD-ILD groups (12 months [IQR: 3–36 months] vs. 12 months [IQR: 4.75–36 months], *p* = 0.815). Comorbidities were more common in non-CTD-ILD patients (70.0% vs. 57.8%), although this difference was not statistically significant (*p* = 0.072). HTN was found to be significantly higher among non-CTD-ILD patients (42.7% vs. 25.6%, *p* = 0.011), while other comorbidities such as DM, IHD, CVA, CKD, CLD, and prior Koch’s disease history did not differ significantly between the groups. Smoking details revealed more smokers in the non-CTD-ILD group (16.4% vs. 7.8%, *p* = 0.068), although this was not statistically significant. Family history of ILD was uncommon and did not differ between these two groups. Treatment patterns differed notably in both groups. Use of only steroids (55.6% vs. 32.7%, *p* = 0.001) and MMF (18.9% vs. 6.4%, *p* = 0.007) was significantly more common in CTD-ILD patients than in non-CTD-ILD patients. Use of NSAIDs, HCQ, DMARDs such as AZA and MTX, and antifibrotics (pirfenidone and nintedanib) did not show statistical differences between the CTD-ILD and non-CTD-ILD groups. Admission rates were significantly higher in non-CTD-ILD patients (38.2% vs. 20.0%, *p* = 0.005), but in-hospital mortality did not significantly differ between the CTD-ILD and non-CTD-ILD groups (13.6% vs. 8.9%, *p* = 0.295). There was a statistically significant difference in HRCT, which showed a predominance of the NSIP pattern in CTD-ILD (53.3% vs. 29.1%, *p* < 0.001), while UIP was more frequent in non-CTD-ILD patients (46.4% vs. 38.9%, *p* < 0.001). All PFT parameters, such as FVC and FEV_1,_ showed no significant differences between the CTD-ILD and non-CTD-ILD groups (*p* > 0.05).

**Table 1:** Baseline demographic, clinical, and radiological parameters among CTD-ILD and non-CTD-ILD patients.

Characteristics	CTD-ILD (*n* = 90)	Non-CTD-ILD (*n* = 110)	*p*-value
Males	18 (20.0%)	45 (40.9%)	**0.002** ^**#**^
Females	72 (80.0%)	65 (59.1%)	
Age at evaluation (years)	49 (36, 58)	57 (47, 68)	**<0.001** ^**#**^
18–40 years	31 (34.4%)	14 (12.7%)	
40–60 years	43 (47.8%)	47 (42.7%)	
>60 years	16 (17.8%)	49 (44.5%)	
Age of onset of ILD (years)	45 (35, 57)	56 (46, 66)	**<0.001** ^**#**^
ILD disease duration (months)	12.00 (3.00, 36.00)	12.00 (4.75, 36.00)	0.815
Comorbidities	52 (57.8%)	77 (70%)	0.072
No comorbidities	38 (42.2%)	33 (30%)	
Any 1 comorbidity	26 (28.9%)	39 (35.5%)	0.324
2 or more comorbidities	18 (20%)	52 (29.1%)	0.14
DM	17 (18.9%)	34 (30.9%)	0.052
HTN	23 (25.6%)	47 (42.7%)	**0.011** ^**#**^
IHD	7 (7.8%)	6 (5.5%)	0.507
CVA	0 (0.0%)	3 (2.7%)	0.32
CKD	2 (2.2%)	4 (3.6%)	0.868
CLD	2 (2.2%)	1 (0.9%)	0.447
Previous history of KOCH’s	16 (17.8%)	19 (17.3%)	0.925
Smokers	7 (7.8%)	18 (16.4%)	0.068
Non-smokers	83 (92.2%)	92 (83.6%)	
Family history			
Yes	2 (2.2%)	7 (6.4%)	0.288
No	88 (97.8%)	103 (93.6%)	
Untreated patients	20 (22.2%)	18 (16.4%)	0.293
Treated patients	70 (77.8%)	92 (83.6%)	
Treatment details			
NSAIDs	46 (51.1%)	65 (59.1%)	0.259
HCQ	6 (6.7%)	6 (5.5%)	0.72
Steroids only	50 (55.6%)	36 (32.7%)	**0.001** ^**#**^
MMF	17 (18.9%)	7 (6.4%)	**0.007** ^**#**^
AZA	7 (7.8%)	2 (1.8%)	0.093
Methotrexate	3 (3.3%)	0 (0%)	0.179
Nintedanib	11 (12.2%)	19 (17.3%)	0.32
Pirfenidone	4 (4.4%)	11 (10.0%)	0.138
Admitted patients	18 (20%)	42 (38.2%)	**0.005** ^**#**^
Non-admitted patients	72 (80%)	68 (61.8%)	
Mortality	8 (8.9%)	15 (13.6%)	0.295
Fibrotic ILDs	79 (87.8%)	90 (81.8%)	0.247
Non-Fibrotic ILDs	11 (12.2%)	20 (18.2%)	
HRCT pattern			
UIP	35 (38.9%)	51 (46.4%)	**<0.001** ^**#**^
NSIP	48 (53.3%)	32 (29.1%)	
Others	7 (7.8%)	27 (24.5%)	
PFT findings			
FVC Act1%	1.39 (1.01, 1.83)	1.38 (0.93, 1.68)	0.341
FVC Act1/Pred	63.40 (43.65, 73.05)	54.15 (40.41, 68.17)	0.097
FEV1 Act1%	1.22 (0.88, 1.61)	1.20 (0.78, 1.41)	0.596
FEV 1%/FVC Act 1%	85.79 (80.56, 91.06)	87.72 (81.87, 91.46)	0.433

All continuous variables are presented as median and interquartile range (25th–75th percentiles) and categorical variables as *n*(%). ^#^Two-tailed *p-*values of <0.05 were considered statistically significant. Pearson’s chi-square test (with continuity correction when cell counts were <5) was used for categorical variables, and the Mann–Whitney *U* test for continuous variables. PFT findings were available only for *n* = 128 ILD patients, as *n* = 72 ILD patients could not perform PFTs. Mortality data reflect deaths during the study period. The significant p values are presented in bold.

### Comparison of serological parameters

Comparison of serological parameters between CTD-ILD and non-CTD-ILD patients is summarized in Table 2. Among fibrosis and ECM remodelling markers, MMP7 levels were significantly lower in CTD-ILD than in non-CTD-ILD (8.69 ng/mL [IQR: 2.42–15.95] vs. 10.28 ng/mL [IQR: 7.36–16.49], *p* = 0.025), while MMP1, MMP9, TIMP1, and CCL18 levels did not differ significantly between groups (*p* = 0.57, *p* = 0.14, *p* = 0.302, and *p* = 0.546, respectively). Among the alveolar epithelial injury markers, KL-6 and SP-D levels showed no significant differences (*p* = 0.256 and *p* = 0.18, respectively). Among endothelial injury markers, LDH levels were found to be elevated in CTD-ILD patients compared to non-CTD-ILD patients (38.76 pg./mL [IQR 31.01–53.66] vs. 34.05 pg./mL [IQR 29.60–47.04], *p* = 0.019), while ICAM did not differ significantly (*p* = 0.887). Among systemic inflammation markers, anti-MDA5 antibody levels were significantly higher in the CTD-ILD group (69.83 pg./mL [IQR: 52.78–80.45] vs. 51.21 pg./mL [IQR 37.95–67.45], *p* = 0.001) than in the non-CTD-ILD group. Other inflammatory mediators, ferritin, CIC-C1q, and ACE, showed no significant differences (*p* = 0.658, *p* = 0.562, and *p* = 0.328, respectively). Cytokines were significantly different between CTD-ILD and non-CTD-ILD patients. Among Th1 cytokines, TNF-α levels (3.78 pg./mL [IQR: 1.95–16.49] vs. 2.87 pg./mL [IQR: 1.60–4.48], *p* = 0.004) and IFN-γ (5.48 pg./mL [IQR: 4.41–8.05] vs. 5.03 pg./mL [IQR: 3.82–6.60], *p* = 0.035) were elevated in CTD-ILD patients than in non-CTD-ILD patients, whereas IL-1β levels did not differ between the two groups (*p* = 0.496). Among Th17 cytokines, IL-22 levels (4.94 pg./mL [IQR: 3.27–48.06] vs. 3.57 pg./mL [IQR: 2.62–6.67] *p* = 0.001) were significantly higher in CTD-ILD patients than in non-CTD-ILD patients, whereas IL-17A levels did not differ (*p* = 0.108). Th2 cytokine: IL-4 levels (7.7 pg./mL [IQR: 5.12–9.85] vs. 6.74 pg./mL [IQR: 4.39–8.53] *p* = 0.026) were significantly higher among CTD-ILD patients than non-CTD-ILD patients.

**Table 2:** Serological parameters among CTD-ILD and non-CTD-ILD patients studied (*n* = 200).

Group	Serological parameters	CTD-ILD (*n* = 90)	Non-CTD-ILD (*n* = 110)	*p-*value
		Median (IQR)	Median (IQR)	
Fibrosis and ECM remodelling	MMP 1 (pg/mL)	5774.11 (2336.51, 14072.51)	6569.34 (2528.41, 12014.56)	0.57
	MMP 7 (ng/mL)	8.69 (2.42, 15.95)	10.28 (7.36, 16.49)	**0.025** ^#^
	MMP 9 (ng/mL)	676.86 (444.99, 867.50)	744.9 (532.86, 873.09)	0.14
	TIMP 1 (ng/mL)	188.92 (114.89, 313.37)	177.68 (106.23, 267.11)	0.302
	CCL 18 (pg/mL)	95,726 (69965.53, 141823.47)	98318.11 (69259.69, 168855.16)	0.546
Alveolar epithelial injury markers	KL-6 (ng/mL)	74.48 (57.23, 117.70)	64.34 (39.64, 100.41)	0.256
	SP-D (pg/mL)	1206.36 (578.08, 2619.48)	1288.94 (831.87, 4125.50)	0.18
Endothelial injury markers	ICAM-1 (ng/mL)	415.66 (285.97, 652.23)	424.76 (257.49, 840.62)	0.887
	LDH (pg/mL)	38.76 (31.01, 53.66)	34.05 (29.60, 47.04)	**0.019** ^#^
Systemic inflammation markers	Ferritin (ng/mL)	77.13 (24.26, 192.00)	73.66 (26.71, 146.13)	0.658
	CIC-C1q (μgEq/mL)	5.96 (3.68, 10.40)	5.36 (3.55, 10.89)	0.562
	ACE (ng/mL)	78.4 (59.05, 110.63)	88.5 (51.26, 158.45)	0.328
	Anti-MDA5Ab (pg/mL)	69.83 (52.78, 80.45)	51.21 (37.95, 67.45)	**0.001** ^#^
Cytokines	IL-1β (pg/mL)	3.91 (1.98, 6.14)	3.53 (2.03, 5.95)	0.496
	TNF-α (pg/mL)	3.78 (1.95, 16.49)	2.87 (1.60, 4.48)	**0.004** ^#^
	IFN-γ (pg/mL)	5.48 (4.41, 8.05)	5.03 (3.82, 6.60)	**0.035** ^#^
	IL-6 (pg/mL)	150.74 (44.77, 473.32)	164.86 (43.64, 506.25)	0.705
	IL-17A (pg/mL)	12.92 (11.09, 15.63)	11.67 (10.20, 15.69)	0.108
	IL − 22 (pg/mL)	4.94 (3.27, 48.06)	3.57 (2.62, 6.67)	**0.001** ^#^
	IL-4 (pg/mL)	7.7 (5.12, 9.85)	6.74 (4.39, 8.53)	**0.026** ^#^

All values were compared between the two groups using the Mann–Whitney *U* test. ^#^A two-tailed *p*-value of <0.05 was considered statistically significant. Anti-MDA5 antibody data were available only for *n* = 150 ILD patients, and imputation was not done as missing data were higher than 25%. The significant p values are presented in bold.

In the multivariable binary logistic regression analysis adjusted for age, sex, HRCT pattern, and hypertension, TNF-α (adjusted OR = 1.012, 95% CI: 1.000–1.024; *p* = 0.043) and IL-22 (adjusted OR = 1.006, 95% CI: 1.000–1.011; *p* = 0.032) remained independent predictors of CTD-ILD diagnosis. IL-4 also showed an independent association with CTD-ILD after multivariable adjustment (adjusted OR = 1.023, 95% CI: 1.000–1.046; *p* = 0.049). IFN-γ, which was significant in the univariate analysis (OR = 1.039, 95% CI: 1.003–1.077; *p* = 0.034), lost statistical significance after multivariable adjustment (*p* = 0.135), suggesting confounding by the included covariates. MMP7, LDH, and anti-MDA5 antibody levels did not demonstrate independent associations with CTD-ILD diagnosis on multivariable analysis (*p* > 0.05 for all). Detailed logistic regression results are presented in Supplementary Table S2.

### ANA IFA titres and IFA patterns in CTD-ILD and non-CTD-ILD patients across HRCT patterns

The distribution of ANA IFA titres and patterns among CTD-ILD and non-CTD-ILD patients across HRCT patterns is presented in Table 3. ANA positivity was observed in 123 ILD patients [61.5% (123/200)]. Out of the total ANA-positive ILD patients (*n* = 123), the ANA positivity rates differed significantly (*p* < 0.001), where 48 patients had ANA high titre (≥1:160) and 75 patients had ANA low titre (<1:160). The proportion of ANA high-titre patients (≥1:160) was higher in CTD-ILD patients than in non-CTD-ILD patients (70.8% (*n* = 34) and 29.2% (*n* = 14), respectively). The proportion of ANA low-titre patients (<1:160) was higher in non-CTD-ILD patients than in CTD-ILD patients (61.3% (*n* = 46) and 38.7% (*n* = 29), respectively). The 14 non-CTD-ILD patients with high ANA titres included 7 IPF patients, 2 fHP patients, 1 iNSIP patient, and 4 ‘other rare ILD’ patients. Among the CTD-ILD and ANA high-titre patient groups, the NSIP pattern [55.9% (*n* = 19)] was more frequent, followed by the UIP pattern [35.3% (*n* = 12)] and other patterns [8.8% (*n* = 3)]. Among the non-CTD-ILD, ANA high-titre patient group, the UIP pattern [78.6% (*n* = 11)] was more frequent, followed by the NSIP pattern [14.3% (*n* = 2)], and other patterns [7.1% (*n* = 1)]. Among the CTD-ILD, ANA low-titre patient group, NSIP, and UIP patterns were equally distributed [48.3% (*n* = 14) and 48.3% (*n* = 14)], followed by other patterns [3.4% (*n* = 1)]. Among the non-CTD-ILD, ANA low-titre patient group, the UIP pattern [45.7% (*n* = 21)] was more frequent, followed by the NSIP pattern [32.6% (*n* = 15)], and other patterns [21.7% (*n* = 10)].

**Table 3:** Distribution of ANA titres and IFA patterns in CTD-ILD vs. non-CTD-ILD across HRCT patterns (*n* = 200).

ANA positivity/IFA pattern	CTD-ILD	CTD-ILD (*n* = 90)			Non CTD-ILD	Non CTD-ILD (*n* = 110)			*p-*value
		UIP	NSIP	Others		UIP	NSIP	Others	
Total ILDs (*n* = 200)	90 (45%)	35 (38.9%)	48 (53.3%)	7 (7.8%)	110 (55%)	51 (46.4)	32 (29.1)	27 (24.5%)	
Total ANA Positives (*n* = 123)	63 (51.2%)	26 (74.3%)	33 (68.8%)	4 (57.1%)	60 (48.8%)	32 (62.7%)	17 (53.1%)	11 (40.7%)	0.025^#^
ANA high titre (≥1:160) (*n* = 48)	34 (70.8%)	12 (35.3%)	19 (55.9%)	3 (8.8%)	14 (29.2%)	11 (78.6%)	2 (14.3%)	1 (7.1%)	
ANA low titre (<1:160) (*n* = 75)	29 (38.7%)	14 (48.3%)	14 (48.3%)	1 (3.4%)	46 (61.3%)	21 (45.7%)	15 (32.6%)	10 (21.7%)	<0.001^#^
Homogeneous (*n* = 13)	8 (61.5%)	2 (25.0%)	6 (75.0%)	0 (0%)	5 (38.5%)	3 (60.0%)	1 (20.0%)	1 (20.0%)	0.431
ANA high titre (≥1:160) (*n* = 5)	5 (100%)	1 (20%)	4 (80%)	0 (0%)	0 (0%)	0 (0%)	0 (0%)	0 (0%)	
ANA low titre (<1:160) (*n* = 8)	3 (37.5%)	1 (33.3%)	2 (66.7%)	0 (0%)	5 (62.5%)	3 (60%)	1 (20%)	1 (20%)	
Nucleolar (*n* = 4)	3 (75%)	2 (66.7%)	0 (0%)	1 (33.3%)	1 (25%)	0 (0%)	0 (0%)	0 (0%)	0.334
ANA high titre (≥1:160) (*n* = 2)	2 (100%)	1 (50%)	0 (0%)	1 (50%)	0 (0%)	0 (0%)	0 (0%)	0 (0%)	
ANA low titre (<1:160) (*n* = 2)	1 (50%)	1 (100%)	0 (0%)	0 (0%)	1 (50%)	0 (0%)	0 (0%)	1 (100%)	
Coarse/fine speckled (*n* = 66)	35 (53%)	17 (48.6%)	15 (42.8%)	3 (8.6%)	31 (47%)	17 (54.8%)	12 (38.7%)	2 (6.5%)	0.665
ANA high titre (≥1:160) (*n* = 29)	21 (72.4%)	9 (42.9%)	10 (47.6%)	2 (9.5%)	8 (27.6%)	7 (87.5%)	1 (12.5%)	0 (0%)	
ANA low titre (<1:160) (*n* = 37)	14 (37.8%)	8 (57.2%)	5 (35.7%)	1 (7.1%)	23 (62.2%)	10 (43.5%)	11 (47.8%)	2 (8.7%)	
Cytoplasmic/mitochondrial (*n* = 38)	15 (39.5%)	6 (40%)	9 (60%)	0 (0%)	23 (60.5%)	12 (52.2%)	4 (17.4%)	7 (30.4%)	0.081
ANA high titre (≥1:160) (*n* = 19)	13 (68.4%)	5 (38.5%)	8 (61.5%)	0 (0%)	6 (31.6%)	4 (66.6%)	1 (16.7%)	1 (16.7%)	
ANA low titre (<1:160) (*n* = 19)	2 (10.5%)	1 (50%)	1 (50%)	0 (0%)	17 (89.5%)	8 (47.1%)	3 (17.6%)	6 (35.3%)	

All values are presented as *n*(%). The percentages of total ANA-positive cases with UIP, NSIP, and other radiological patterns were calculated using the total counts of UIP, NSIP, and other radiological patterns in the CTD-ILD and non-CTD-ILD groups. The percentages of high- and low-titre ANA-positive cases under the UIP, NSIP, and other radiological patterns for each ANA pattern were calculated from the total high- and low-titre ANA-positive cases, respectively, for each ANA pattern in the CTD-ILD and non-CTD-ILD groups. Comparisons of ANA IFA patterns were performed between the CTD-ILD and non-CTD-ILD groups using Pearson’s chi-square test; analyses were not stratified by HRCT subtypes due to small numbers within each category. ^#^A two-tailed *p-*values of <0.05 were considered statistically significant.

Among ILD patients with ANA positivity (*n* = 123), 13 (10.6%) had a homogeneous pattern, 4 (3.3%) had a nucleolar pattern, 66 (56.7%) had a fine speckled pattern, and 38 (30.9%) had a cytoplasmic/mitochondrial pattern. A homogeneous pattern was more frequent in CTD-ILD patients than in non-CTD-ILD patients (61.5% vs. 38.5%). The nucleolar pattern was more frequent in CTD-ILD patients than in non-CTD-ILD patients (75% vs. 25%). The fine speckled pattern was slightly higher in CTD-ILD patients than in non-CTD-ILD patients (53% vs. 47%), and the cytoplasmic or mitochondrial pattern was higher among non-CTD-ILD patients than in CTD-ILD patients (60.5% vs. 39.5%). The distribution of the HRCT patterns within the CTD-ILD and non-CTD-ILD groups for ANA IFA patterns is presented in Table 3.

### Distribution of ANA positivity and ANA IFA patterns among CTD-ILD subgroups

**Figure 1:**
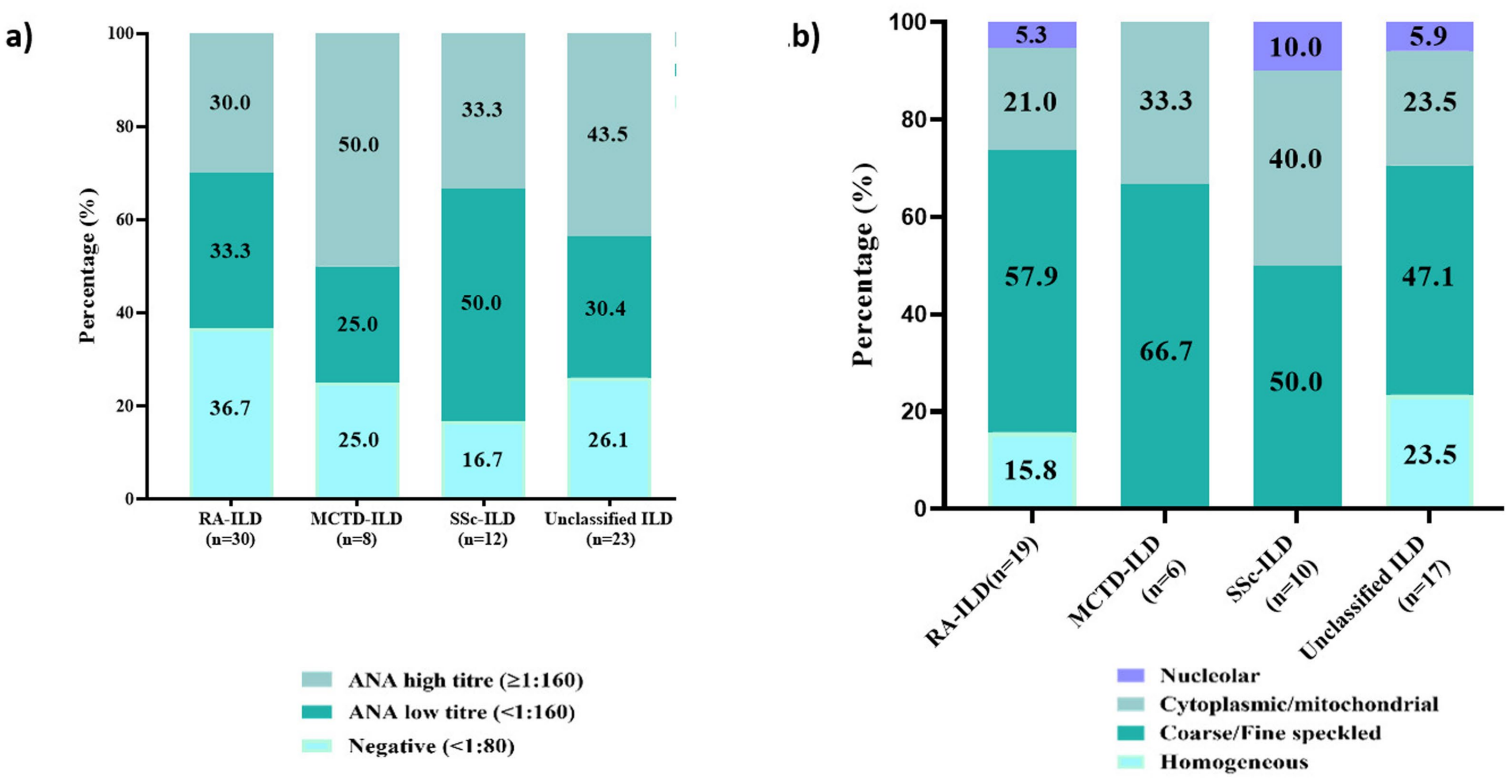
Distribution of antinuclear antibodies (ANA) IFA titres and patterns in CTD-ILD subgroups. **(a)** A stacked bar chart illustrating the proportional distribution of ANA titres among the four CTD-ILD subtypes: RA-ILD, MCTD-ILD, SSc-ILD, and unclassified ILD. ANA titres are categorized as high titre (≥1:160), low titre (<1:160), and negative (<1:80). **(b)** A stacked bar chart illustrating the proportional distribution of ANA IFA patterns among four CTD-ILD subtypes: RA-ILD, MCTD-ILD, SSc-ILD, and unclassified ILD.

Among the CTD-ILD patients, 30 patients (33.3%) had RA-ILD, followed by 23 patients (25.6%) with unclassified CTD-ILD, 12 patients (13.3%) with SSc-ILD, and 8 patients (8.9%) with MCTD-ILD. As shown in Figure 1a, a higher percentage of ANA positivity with high titre (≥1:160) was noted in MCTD-ILD (50.0%), unclassified ILD (43.5%), and SSc-ILD (33.3%); whereas in RA-ILD, a lower percentage of ANA positivity with high titre was noted (30.0%).

**Figure 2:**
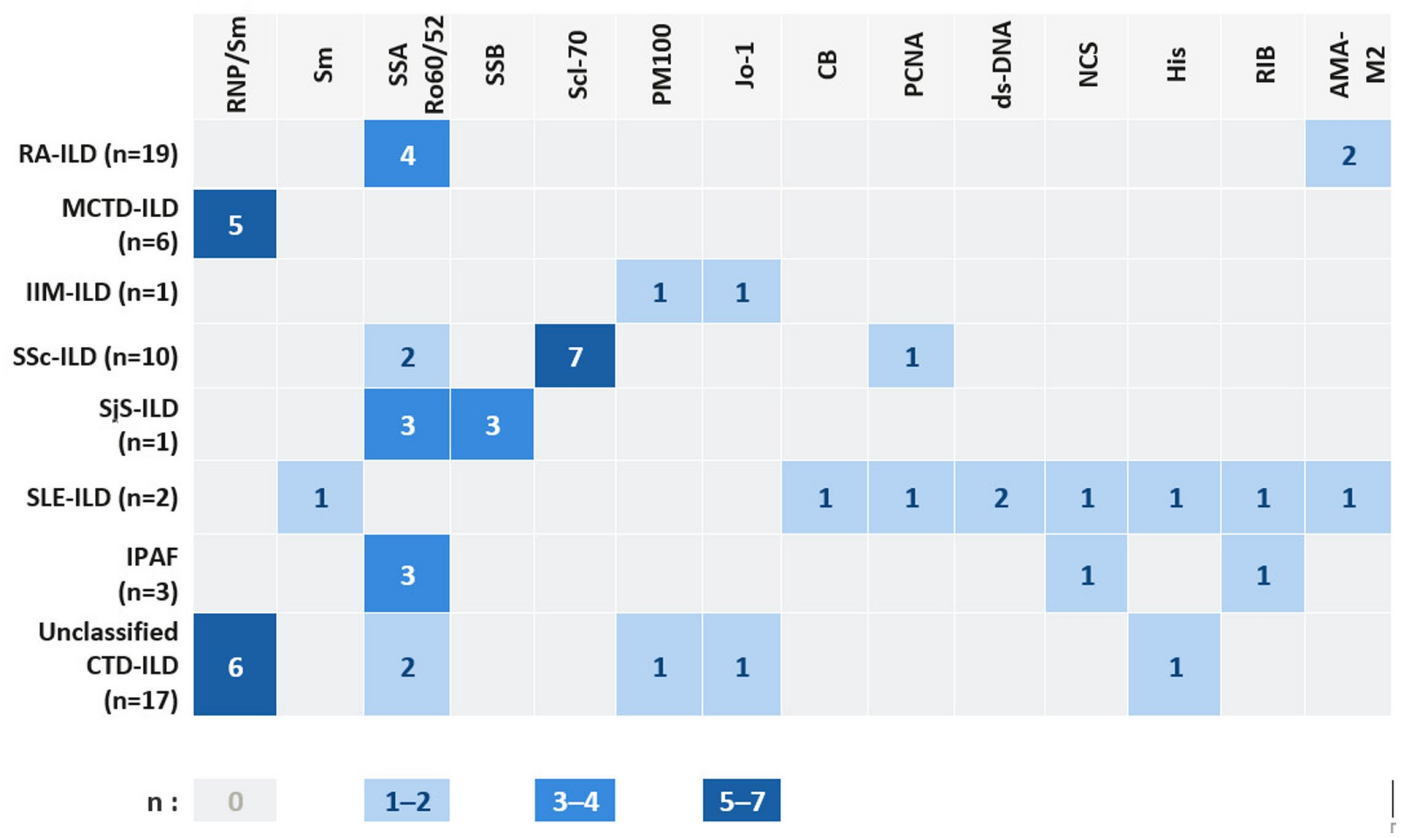
Distribution of ANA specificities by line blot assay (LIA) among ANA-positive CTD-ILD patients. Figure represents a heatmap on the basis of the number of autoantibody positive cases in CTD-ILD subtypes. Each cell shows the number of ANA-positive patients (*n* = 63) with a given antibody specificity within each CTD-ILD subtype. RA, Rheumatoid arthritis; MCTD, Mixed connective tissue disease; IIM, Idiopathic inflammatory myopathy; SSc, Systemic sclerosis; Sjögren’s syndrome; SLE, Systemic lupus erythematosus; IPAF, Interstitial pneumonia with autoimmune features; CTD, Connective tissue disease; ILD, Interstitial lung disease. Autoantibodies: RNP/Sm = U1-ribonucleoprotein/Smith; SSA, Sjögren’s syndrome antigen A (Ro60/52); SSB, Sjögren’s syndrome antigen B (La); Scl-70, anti-topoisomerase I; PM100, polymyositis-100; Jo-1, anti-histidyl tRNA synthetase; CB, Chromatin binding; PCNA, Proliferating cell nuclear antigen; dsDNA, double-stranded DNA; NCS, Nucleosomes; His, Histones; RIB, Ribosomal P protein; AMA-M2, Anti-mitochondrial antibody M2.

As shown in Figure 1b, among the RA-ILD patients with ANA positivity (*n* = 19), 57.9% had a fine speckled pattern, 21.0% had a cytoplasmic/mitochondrial pattern, 15.8% had a homogeneous pattern, and 5.3% had a nucleolar pattern. Among MCTD-ILD patients with ANA positivity (*n* = 6), 66.7% had a fine speckled pattern, and the remaining 33.3% of patients had a cytoplasmic/mitochondrial pattern. Among SSc-ILD patients with ANA positivity (*n* = 10), 50% had a fine speckled pattern, 40% had a cytoplasmic/mitochondrial pattern, and 10% had a nucleolar pattern. Among unclassified ILDs with ANA positivity (*n* = 17), the distribution of ANA IFA patterns was 23.5, 47.1, 23.5, and 5.9% for homogeneous, fine speckled, cytoplasmic or mitochondrial, and nucleolar patterns, respectively. An IIM-ILD patient with a high ANA titre (*n* = 1) had a fine speckled pattern, a Sjögren’s-ILD patient with a high ANA titre (*n* = 1) had a fine speckled pattern, and SLE-ILD patients with a high ANA titre (*n* = 2) had an equal distribution of homogeneous and cytoplasmic patterns. IPAF patients with high ANA positivity (*n* = 2) had an equal distribution of homogeneous and fine speckled patterns, whereas one IPAF patient with low ANA positivity had a fine speckled pattern. Further, the distribution of ANA specificities for high-titre ANA-positive CTD-ILD patients is shown in Figure 2.

### Correlation of demographic, clinical, and PFT parameters with serological parameters

**Figure 3:**
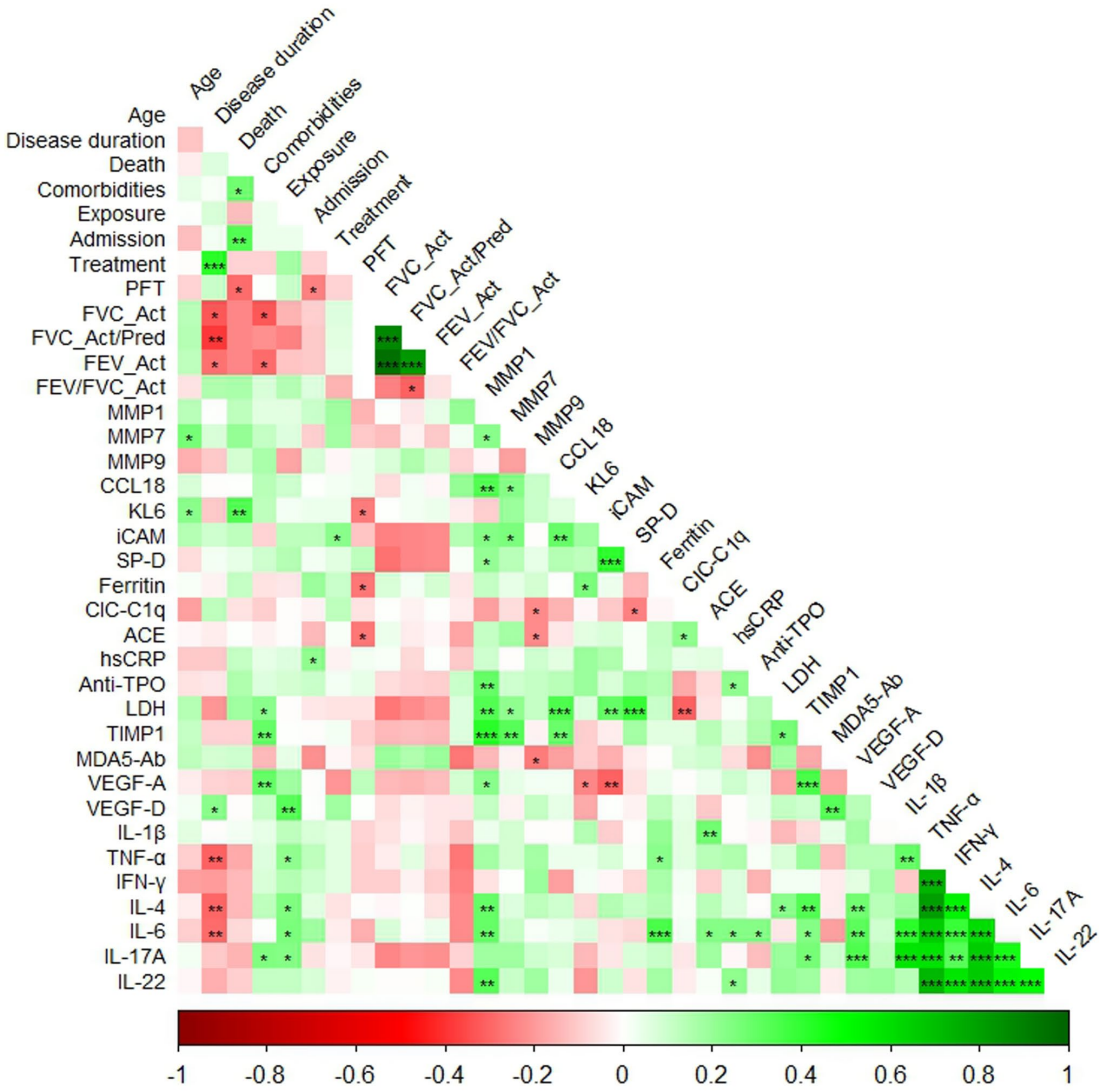
Correlation matrix of serological parameters with demographic, clinical, and PFT parameters in patients with CTD-ILD. The correlation matrix with Spearman’s correlation coefficients of all serum parameters with PFT and demographic details studied in patients in the CTD-ILD group. The color scale ranges from −1 (red, strong negative correlation) to +1 (green, strong positive correlation), with white indicating no correlation. Asterisks indicate statistical significance: *p* < 0.05 (*), *p* < 0.01 (**), and *p* < 0.001 (***).

As shown in Figure 3, among fibrosis and ECM remodelling markers, MMP7 showed a positive correlation with age (*p* < 0.05), while TIMP-1 was positively correlated with comorbidity burden (*p* < 0.05). Among the alveolar epithelial injury markers, KL-6 was positively correlated with both age and mortality (*p* < 0.05). Among the endothelial and cellular injury markers, LDH was positively correlated with comorbidities, and ICAM showed a positive correlation with treatment (*p* < 0.05). Among systemic inflammation markers, ferritin and ACE demonstrated negative correlations with the ability to perform PFT (*p* < 0.05).

Among the cytokines, a strong and consistent positive correlation was observed across the Th1, Th17, and Th2 cytokine axes, encompassing IL-1β, TNF-α, IFN-γ, IL-4, IL-6, IL-17A, and IL-22 (*p* < 0.05). TNF-α, IL-4, and IL-6 levels showed significant negative correlations with disease duration (*p* < 0.05). Conversely, TNF-α, IL-6, IL-17A, and IL-22 levels were significantly positively correlated with exposure history (*p* < 0.05). Mortality among CTD-ILD patients was significantly positively correlated with comorbidity burden and hospital admission (*p* < 0.05).

### Hospital admissions and mortality among patients with CTD-ILD

As shown in Table 4, among 90 CTD-ILD patients, 18 (20.0%) required hospitalization during the study period. Admitted and non-admitted patients were comparable in age, sex, disease duration, and treatment status (*p* > 0.05 for all). Mortality was significantly higher among admitted patients than among non-admitted patients (27.8% vs. 4.2%; *p* = 0.007), with 8 CTD-ILD patients (8.9%) dying during the study period, accounting for 34.8% of the total ILD-related mortality in the cohort. Admitted patients demonstrated a higher, though statistically non-significant (p > 0.05), comorbidity burden, with diabetes mellitus (33.3% vs. 15.3%), ischaemic heart disease (16.7% vs. 5.6%), chronic kidney disease (11.1% vs. 0%), and chronic liver disease (11.1% vs. 0%) more frequently observed among hospitalized patients. The ability to perform PFT was significantly lower in admitted patients (33.3% vs. 63.9%; *p* = 0.019). Radiologically, the UIP pattern was more prevalent among admitted patients (50.0% vs. 36.1%), although this difference did not reach statistical significance (*p* = 0.551).

**Table 4:** Demographic and clinico-radiological parameters among admitted and non-admitted CTD-ILD patients (*n* = 90).

Characteristics	Admitted (*n* = 18)	Non-admitted (*n* = 72)	*p-*values
Male	3 (16.67%)	15 (20.83%)	0.947
Female	15 (83.33%)	57 (79.17%)	
Age at evaluation (years)	54 (42, 63)	53 (42, 63)	0.244
18–40 years	9 (50.0%)	22 (30.6%)	
40–60 years	6 (33.3%)	37 (51.4%)	
>60 years	3 (16.7%)	13 (18.1%)	
Age of onset of ILD (years)	52 (41, 63)	52 (41, 63)	0.181
ILD disease duration (months)	12.00 (4.00, 36.00)	12.00 (4.00, 36.00)	0.847
Comorbidities	10 (55.6%)	34 (47.2%)	0.749
No comorbidities	8 (44.4%)	38 (52.8%)	
Any 1 comorbidity	5 (27.8%)	21 (29.2%)	0.907
2 or more comorbidities	5 (27.8%)	13 (18.1%)	0.553
DM	6 (33.3%)	11 (15.3%)	NA
HTN	4 (22.2%)	19 (26.4%)	
IHD	3 (16.7%)	4 (5.6%)	
CVA	0 (0.0%)	0 (0.0%)	
CKD	2 (11.1%)	0 (0.0%)	
CLD	2 (11.1%)	0 (0.0%)	
Previous history of KOCH’s	4 (22.2%)	12 (16.7%)	
Smokers	1 (5.6%)	6 (8.3%)	NA
Non-smokers	17 (94.4%)	66 (91.7%)	
Family History			
Yes	0 (0.0%)	2 (2.8%)	
No	18 (100%)	70 (97.2%)	
Untreated patients	5 (27.8%)	15 (20.8%)	0.751
Treated patients	13 (72.2%)	57 (79.2%)	
Mortality	5 (27.8%)	3 (4.2%)	**0.007** ^**#**^
Fibrotic ILDs	15 (83.3%)	64 (88.9%)	0.809
HRCT pattern			
UIP	9 (50.0%)	26 (36.1%)	0.551
NSIP	8 (44.4%)	40 (55.6%)	
Others	1 (5.6%)	6 (8.3%)	
PFT (able to perform)	6 (33.3%)	46 (63.9%)	**0.019** ^**#**^

All the continuous variables were presented as median (IQR), and categorical variables were presented as *n* (%). The significant *p* values are presented in bold.

^#^A two-tailed *p*-values of <0.05 were considered statistically significant.

## Discussion

In routine practice, distinguishing autoimmune-driven ILD from idiopathic or exposure-related fibrosing ILD remains a major diagnostic challenge, particularly in India, where extended autoantibody panels and multiplex assays are not widely available (1). The present study compared autoantibody profiles, ANA titres, IFA patterns, and comprehensive serum parameter profiles between CTD-ILD and non-CTD-ILD patients from Western India. This comparative design was chosen because CTD-ILD and non-CTD-ILD, including IPF, fibrotic NSIP, and fibrotic HP, often share overlapping clinical and radiological features yet differ fundamentally in their pathobiology and treatment modalities (1).

The present study reported a higher proportion of CTD-ILD (45%) compared to Indian studies reporting 4.5–23.0% and global cohorts reporting 7.5–34.8% among all ILDs (2, 19, 20, 26). This likely reflects the multidisciplinary setting of our tertiary centre, where pulmonologists, rheumatologists, radiologists, pathologists, and an autoimmune research laboratory facilitated comprehensive evaluation, reducing the likelihood of missed CTD-ILD diagnoses (27). The female preponderance and younger age at evaluation among CTD-ILD patients in the present study reflected the earlier onset of CTDs, predisposing these patients to ILD at a younger age, similar to other Indian and global studies (19, 21, 26, 28, 29). In contrast, non-CTD-ILDs, such as IPF and fHP, typically manifest later in adulthood and with male predominance (22, 30, 31). In the present study, systemic hypertension was more frequent among non-CTD-ILD patients, consistent with previous reports of a higher cardiovascular comorbidity burden in IPF and fibrotic HP, whereas CTD-ILD patients presented more often with autoimmune comorbidities. The EMPIRE registry reported cardiovascular diseases in 73.9% of IPF patients, with arterial hypertension as the most frequent comorbidity (53%). Prior et al. (34) similarly reported hypertension as the leading comorbidity in fibrotic HP (55.5%) (32–34).

Oliveira et al. reported that steroid monotherapy and MMF were used more frequently in CTD-ILD patients, a finding consistent with this study (21). These findings were consistent with expert reviews and recent guidelines, which recommend MMF as the preferred agent across CTD-ILD subtypes due to its favourable safety and efficacy (35–38). Steroids remain more common in RA-ILD and other CTD-ILDs and are recommended in IIM-ILD. Although guidelines have suggested combining antifibrotics with immunosuppressants in the treatment of CTD-ILD, in this study, antifibrotics, such as nintedanib and pirfenidone, were used more often in non-CTD-ILD patients (39).

In our cohort, the NSIP pattern emerged as the most frequent radiological finding among CTD-ILD patients, while UIP was the leading pattern in non-CTD-ILD, consistent with a systematic review of 122 studies including 8,266 CTD-ILD patients, which identified fibrotic NSIP as the predominant pattern (27–76%), except in RA-ILD, where UIP was more common (46%) (3, 40). Given that RA-ILD accounted for one-third (33.3%) of the CTD-ILD cases in this study, the higher proportion of UIP within this group explained why UIP was the second most common pattern overall in CTD-ILD. The updated 2025 classification guidelines for interstitial pneumonia also identified CTD as the most common cause of NSIP (1).

This study demonstrated higher ANA positivity in CTD-ILD than in non-CTD-ILD, with a predominance of high-titre ANA and IFA speckled patterns. These findings were consistent with international cohorts reporting ANA positivity in 55–65% of CTD-ILD, particularly high-titre ANA in SSc-ILD (41). Importantly, stratification of ANA IFA patterns by HRCT subtype revealed greater ANA activity in NSIP than in UIP, reflecting the higher ANA positivity rates of SSc-ILD, MCTD-ILD, and IIM-ILD, which predominantly present with NSIP, compared to RA-ILD, which more commonly presents with UIP and carries relatively lower ANA positivity rates. Subgrouping of CTD-ILD revealed that RA-ILD was enriched for anti-SSA/Ro and AMA-M2 autoantibodies, MCTD-ILD for anti-RNP/Sm autoantibodies, and SSc-ILD for anti-Scl70 autoantibodies. This was also similar to the global ENA distributions (42). ANA positivity in unclassified CTD-ILD was higher in this study (27%) than in European IPAF cohorts (15–20%). This suggests a broader autoantibody spectrum among Indian CTD-ILD patients.

Beyond autoantibody profiling, the present study demonstrated an immune-dominant serological profile in Indian CTD-ILD patients. Anti-MDA5 levels were higher in the CTD-ILD group in the present study, similarly supporting the importance of anti-MDA5 autoantibodies as useful markers of immune-mediated lung injury beyond classic dermatomyositis (43–45). In this study, elevated LDH levels in CTD-ILD patients supported its role as a clinically relevant marker of immune-mediated pulmonary cellular damage. A similar finding was reported by Cheng et al. (46), in which higher LDH levels increased short-term mortality in CTD patients with inflammatory lung injury. Okamoto et al. (47) identified baseline LDH levels as predictive of pneumomediastinum in CTD-ILD under corticosteroid therapy (46, 47). In this study, on multivariable regression analysis, Th1 cytokines (TNF-α and IFN-γ), Th2 cytokine IL-4, and Th17 cytokine IL-22 were higher in CTD-ILD, indicating a mixed Th1/Th2/Th17-skewed milieu associated with immune activation and downstream fibrosis. Contemporary mechanistic reviews have emphasized this loop of immunity-to-fibrosis in CTD-ILD, where cytokine signalling sustains epithelial–mesenchymal crosstalk and amplifies parenchymal injury; our findings fit this paradigm (48–50). The present study also showed lower MMP7 levels in CTD-ILD patients than in the non-CTD-ILD group, consistent with its stronger association with fibrotic remodelling in IPF disease (51).

Interestingly, in this study, the mortality rates were comparable between the CTD-ILD and non-CTD-ILD groups; however, the admission rates were significantly lower in the CTD-ILD group. This suggests that immunosuppressive therapy may have conferred a protective effect against hospitalization. However, hospitalization in CTD-ILD clearly marked a turning point in the disease trajectory, with admitted patients showing higher mortality, greater comorbidity burden, and more severe disease features than non-hospitalized patients. Admitted CTD-ILD patients were enriched with comorbidities such as diabetes and ischaemic heart disease. Only a few admitted patients could undergo PFTs due to worsened disease. Radiologically, the predominance of the UIP pattern reflected advanced disease with poorer prognosis in admitted CTD-ILD patients. These findings were similar to international and other Indian reports, emphasizing that hospitalization in CTD-ILD represents a critical stage of progression, where comorbidities, pulmonary functional decline, and fibrotic radiological patterns converge to drive increased mortality (21, 51).

The findings of this study further highlight the value of detailed ANA IFA pattern recognition as an effective screening tool in resource-limited settings and the value of serological markers as predictors of CTD-ILD to differentiate from non-CTD-ILD. This study also had a few limitations, such as the need for a larger multicentre cohort to further validate the impact of autoantibodies among CTD-ILD subtypes across the country and to explore their association with disease severity. Additionally, the myositis autoantibody panel would have helped categorize unclassified CTD-ILDs in the present study. Future efforts to promote multidisciplinary care models are needed to identify and triage patients at high risk of complications related to CTD-ILD. Such observations will provide insights into the development of international treatment algorithms and guidelines for patients with CTD-ILD.

## Conclusion

This study provides insights into the clinical and autoantibody spectrum of CTD-ILD in Western India and reveals its impact on autoantibody profiling on diagnostic utility and its importance in differentiating CTD-ILD patients from non-CTD-ILD patients. These findings offer additional information on demographics, ANA titres, IFA patterns with disease-specific autoantibody profiles based on radiological patterns, and serological profiles of CTD-ILD patients from India. Future multicentre studies with longitudinal follow-up are warranted to validate and integrate these findings into clinical decision-making.

## Data Availability

The raw data supporting the conclusions of this article will be made available by the authors, without undue reservation.
