# Supplementary material for: Spectrum of autoantibodies and other serological parameters in connective tissue disease–associated interstitial lung disease patients
Source: Front Med (Lausanne). 2026 Jun 3;13:1812588. doi: 10.3389/fmed.2026.1812588 (PMC13271993; doi:10.3389/fmed.2026.1812588)
Supplement: Supplementary file 1 [file Table_1.docx]

**Spectrum of autoantibodies and other serological parameters among connective tissue disease-associated Interstitial Lung Disease (CTD-ILD) patients**

Pooja Jaiswal^2#^, Tanya Athavale^1#^, Amita Athavale^1*^, Hemangini Thakkar^6^, Ridi Khatri^2^, Amrutha Jose^3^, Trisha Samant^2^, Tanaya Tipnis^2^, Namrata Neman^2^, Milind Nadkar^4^, Anjali Rajadhyaksha^4^, Manisha Madkaikar^2^, Vandana Pradhan^2*^

**Online supplementary data**

**Table S1: Percentage of missingness in the values for the serological parameters in CTD-ILD and non-CTD-ILD patients**

| **Serological Parameters** | **CTD-ILD (n=90)** | | **non-CTD-ILD (n=110)** | |
| --- | --- | --- | --- | --- |
|  | **Missingness** | | **Missingness** | |
|  | **N** | **Percent** | **N** | **Percent** |
| MMP 1 | 5 | 5.6 | 7 | 6.4 |
| MMP 7 | 9 | 10 | 17 | 15.5 |
| MMP 9 | 3 | 3.3 | 5 | 4.5 |
| TIMP 1 | 11 | 12.2 | 14 | 12.7 |
| CCL 18 | 3 | 3.3 | 7 | 6.4 |
| KL-6 | 3 | 3.3 | 8 | 7.3 |
| SPD | 2 | 2.2 | 8 | 7.3 |
| iCAM | 8 | 8.9 | 10 | 9.1 |
| LDH | 16 | 17.8 | 21 | 19.1 |
| Ferritin | 4 | 4.4 | 6 | 5.5 |
| CIC-C1q | 4 | 4.4 | 2 | 1.8 |
| ACE | 3 | 3.3 | 0 | 0 |
| Anti-MDA5Ab | 28 | 31.1 | 37 | 33.6 |
| IL-1β | 2 | 2.2 | 3 | 2.7 |
| TNF-α | 2 | 2.2 | 3 | 2.7 |
| IFN-γ | 2 | 2.2 | 3 | 2.7 |
| IL-6 | 2 | 2.2 | 3 | 2.7 |
| IL-17A | 15 | 16.7 | 15 | 13.6 |
| IL -22 | 2 | 2.2 | 3 | 2.7 |
| IL-4 | 2 | 2.2 | 3 | 2.7 |

Table S1: Percentage of missing data for serological parameters in CTD-ILD and non-CTD-ILD patients. The table presents the number (n) and percentage of missing values for each serological parameter across CTD-ILD (n=90) and non-CTD-ILD (n=110) patient groups.

**Table S2: Logistic regression analysis of the significant serological parameters among CTD-ILD and non-CTD-ILD patients studied (n=200)**

| **Group** | **Serological Parameters** | **Univariate analysis** | | | **Multivariate analysis** | | |
| --- | --- | --- | --- | --- | --- | --- | --- |
|  |  | **Odds ratio** | **C.I** | **Unadjusted p value** | **Odds ratio** | **C.I** | **Adjusted p values** |
| Fibrosis and ECM remodelling | MMP7 | 0.068 | 0.941- 1.002 | 0.068 | 0.985 | 0.952- 1.019 | 0.389 |
| Endothelial injury markers | LDH | 1.002 | 0.993- 1.011 | 0.680 | 1.005 | 0.995- 1.015 | 0.309 |
| Systemic inflammation markers | Anti-MDA5Ab | 1.005 | 0.996- 1.013 | 0.277 | 1.007 | 0.998- 1.016 | 0.116 |
| Cytokines | TNF-α | 1.010 | 1.000- 1.020 | **0.054^#^** | 1.012 | 1.000- 1.024 | **0.043^#^** |
|  | IFN-γ | 1.039 | 1.003- 1.077 | **0.034^#^** | 1.028 | 0.991- 1.066 | 0.135 |
|  | IL-22 | 1.007 | 1.002- 1.011 | **0.007^#^** | 1.006 | 1.000- 1.011 | **0.032^#^** |
|  | IL-4 | 1.014 | 0.995- 1.033 | 0.161 | 1.023 | 1.000- 1.046 | **0.049^#^** |

Table S2: Results are expressed as odds ratios (OR) with 95% confidence intervals (CI). In multivariate analysis, p-values are adjusted for age, sex, HRCT patterns, and hypertension. ^#^Two-tailed p values <0.05 were considered statistically significant.
